# Antibody responses in blood and saliva post COVID-19 bivalent booster do not reveal an Omicron BA.4/BA.5- specific response

**DOI:** 10.3389/fimmu.2024.1401209

**Published:** 2024-05-15

**Authors:** Ryan Baker, Rebecca Lawlor, Maeve Smith, Jessica Price, Ashley Eaton, Andrew Lover, Dominique Alfandari, Peter Reinhart, Kathleen F. Arcaro, Barbara A. Osborne

**Affiliations:** ^1^ Department of Veterinary and Animal Sciences, College of Natural Science, University of Massachusetts Amherst, Amherst, MA, United States; ^2^ Institute for Applied Life Sciences (IALS) Clinical Testing Center (ICTC), University of Massachusetts Amherst, Amherst, MA, United States; ^3^ Department of Biostatistics and Epidemiology, School of Public Health and Health Sciences, University of Massachusetts Amherst, Amherst, MA, United States; ^4^ Institute for Applied Life Sciences (IALS), University of Massachusetts Amherst, Amherst, MA, United States

**Keywords:** COVID - 19, antibody response, ELISA, neutralization assay, saliva, dried blood spot cards

## Abstract

**Introduction:**

Current SARS-CoV-2 strains continue to mutate and attempt to evade the antibody response elicited by previous exposures and vaccinations. In September of 2022, the first updated SARS-CoV-2 vaccines, designed to create immune responses specific for the variants circulating in 2022, were approved. These new vaccines, known commonly as the bivalent boost(er), include mRNA that encodes both the original Wuhan-Hu-1 spike protein as well as the spike protein specific to the Omicron BA.4 and BA.5 variants.

**Methods:**

We recruited volunteers from University of Massachusetts student, faculty and staff members to provide samples of blood and saliva at four different time points, including pre-boost and three times post boost and analyzed samples for antibody production as well as neutralization of virus.

**Results:**

Our data provide a comprehensive analysis of the antibody response following a single dose of the bivalent boost over a 6-month period and support previous findings that the response induced after the bivalent boost does not create a strong BA.4/BA.5-specific antibody response.

**Conclusion:**

We found no evidence of a specific anti-BA.4/BA.5 response developing over time, including in a sub-population of individuals who become infected after a single dose of the bivalent booster. Additionally, we present data that support the use of saliva samples as a reliable alternative to blood for antibody detection against specific SARS-CoV-2 antigens.

## Introduction

In fall 2022, several companies including Pfizer/BioNTech and Moderna produced updated SARS-CoV-2 mRNA vaccines containing the mRNA spike proteins of the major circulating variants of concern in 2022 (Omicron BA.4 and BA.5). These updated vaccines contain mRNA for both the original Wuhan-Hu-1 and the Omicron BA.4/BA.5 variants and were approved by the FDA in August of 2022^1^,^2^. Preliminary studies indicated that the effectiveness of bivalent boosters was higher than that of original monovalent boosters at decreasing incidences of severe COVID-19 (1), but several subsequent studies reported that the antibody response resulting after the bivalent boost is not specific to the newer variants (2–6). Given that this bivalent boost was approved in late 2022, the IgG response to this bivalent booster over an extended period is largely unstudied. Additionally, the antibody response resulting after infection in individuals who previously received the bivalent booster remains unknown. To address these issues, we designed a survey of individuals both pre-boost and at several time points post-boost to determine serum IgG responses to both the original Wuhan-Hu-1 and Omicron BA.4/BA.5 variants.

Another goal of our study was to design a strategy to collect reliable data while minimizing the use of invasive protocols. We present data that contain a dried blood spot (DBS) collection method which minimizes invasive techniques compared to typical blood collection methods (7–10). As an alternative to blood, we also evaluated the antibody response in saliva. Salivary IgG is known to be indicative of IgG in circulation, including IgG for SARS-CoV-2 (11–14). Our findings indicate that saliva is a reliable alternative to DBS collection. The use of saliva will likely maximize participation in epidemiological and immunological studies due to the simpler and less painful sample collection method.

## Materials and methods

### Recruitment of participants

Between October 2022 and January 2023 participants were recruited to participate in an IRB-approved study at the University of Massachusetts-Amherst (UMass-Amherst IRB # 3852, October 11, 2022). Recruitment relied on group emails, flyers, and word of mouth. Participants were eligible if they planned on receiving a bivalent booster and were willing to provide dried blood spot (DBS) and saliva samples at four timepoints; Pre = prior to receiving the bivalent boost, Post 1 = 2–3 weeks after receiving the bivalent boost, Post 2 = 3 months of after receiving the bivalent boost, and Post 3 = 6 months after receiving the bivalent boost. At the 2–3 week, Post 1, timepoint participants were asked to complete a brief demographic and health questionnaire, which included questions about infection and vaccination history. The questionnaire was asked at this time to include symptoms experienced after receiving the bivalent boost. At the 6-months post-boost timepoint, Post 3, participants were asked to complete a questionnaire regarding positive COVID-19 tests since Post 1. Questionnaires were sent as surveys via the HIPAA-compliant Research Electronic Data Capture (REDCap).

### Sample collection

Participants were given directions to the Institute of Applied Life Sciences at the University of Massachusetts Amherst, where a room was dedicated every business day for participants to provide samples. After donating the Pre bivalent boost samples, participants were asked to return to the collection room 2–3-weeks after receiving the boost (Post 1), 3 months after receiving the boost (Post 2), and 6 months after receiving the boost (Post 3). Blood samples were collected with a McKesson Safety Lancet (Cat. No. 366594). Blood was allowed to flow onto the five, 1.27 cm circles of a Whatman protein saver card (Cat. No. 10534612) to create the dried blood spot(s) (DBS). After an overnight drying period at room temperature, the DBS samples were placed into plastic bags and stored at 4°C. In addition to DBS, participants were asked to provide saliva. Participants were given water to rinse the mouth of solids and then were asked to donate 2 mL of saliva into a 5 mL container over a 10-minute time interval. Saliva samples were immediately frozen at -20°C until further processing and running of immunoassays.

### Preparing DBS and saliva for immunoassays

For all ELISAs conducted on the DBS samples, a 6 mm disc of dried blood was cut from the protein saver card and incubated in 500 µL 1X Tris buffered saline with Tween (TBST) overnight, shaking, at 4°C. Estimating that the amount of dried blood on the 6 mm DBS disc is around 12.5 µL, the resulting blood eluate began at a dilution of 1:40 (12.5 µL dried blood in 500 µL solution). For the cell-based pseudo-virus neutralization assays, the blood eluate sample preparation mirrored that of the ELISA preparation also resulting in a 1:40 dilution, but Dulbecco’s Modified Eagle Medium (DMEM) was used to form the starting eluate instead of TBST. Starting with the frozen saliva samples, the saliva was thawed, heat inactivated at 56°C for 10 minutes, and centrifuged at 6000 g for 10 minutes. The resulting supernatant was aliquoted and stored at -20°C until samples were thawed once again, just before being included in immunoassays.

### SARS-CoV-2 RBD enzyme linked immuno-sorbent assays

Anti-Wuhan-Hu-1 receptor binding domain (RBD) and anti-Omicron BA.4/BA.5 RBD antibodies were measured using enzyme-linked immunosorbent assays (ELISAs). 96 well flat-bottom immuno plates (Thermo Sci., Cat. No. 442404) were coated with 50 µL of respective RBD proteins at a concentration of 1 µg/mL in phosphate buffered saline (PBS). RBD proteins were purchased from R&D Systems (Cat. No. 10500-CV and 11229-CV). Plates were incubated overnight, shaking, at 4°C. The following morning, unbound RBD/PBS was flicked from the plate followed by blocking with 5% non-fat dried milk (NFDM) purchased from VWR Life Sciences (Cat. No. 97063–958) in TBST (200 µL per well) for 1 hour at room temperature while shaking. The blocking step was followed by washing with 200 µL TBST per well three times. Blood eluate samples (50 µL) were then added at various dilutions. Due to differences in antibody levels among individuals, dilutions ranged from 1:40 (original eluate) to 1:8000 diluted in TBST to calculate accurate concentrations of all samples. Saliva supernatant was added in dilutions ranging from undiluted saliva supernatant to 1:100 diluted in TBST. Human IgG or IgA (InVivogen, Cat. No. srbd-mab1 or srbd-mab6) known to bind Wuhan-Hu-1 RBD were included starting at 2 µg/mL and diluted 1:4 serially to generate standard curves. All samples and standards were plated in duplicates and added alongside 50 µL 2% NFDM in TBST. Samples were incubated for 2 hours at RT shaking. Samples/NFDM were discarded followed by a wash. Secondary antibodies, anti-human IgG or anti-human IgA conjugated with horseradish peroxidase (Jaxon Immuno., Cat. No. 309–035-003 and 309–035-011) were then added in 50 µL volumes at a 1:1000 dilution in TBST to each well for the detection of anti-SARS-CoV-2 RBD IgG or IgA respectively. Plates were incubated for 1 hour shaking at RT and the secondary antibodies were discarded followed by another wash. 2,2’-azino-bis [3-ethylbenzothiazoline-6-sulfonic acid] diammonium salt (ABTS, Sigma Aldrich, Cat. No. 11112422001), was dissolved in 100 mM sodium acetate for a final stock concentration of 0.2 mg/mL, catalyzed with hydrogen peroxide (1 µL/mL), and added to each well (100 µL). Plates were incubated for 25 minutes at 37°C and absorbance was read at 405 nm.

### Total antibody ELISA

ELISAs were used to determine the concentration of total IgG in saliva samples which had excess aliquots. The protocol for total IgG detection mirrored that of the SARS-CoV-2 RBD ELISAs, however the wells were coated with anti-human antibodies instead of RBD. For total IgG detection, plates were coated with 1 µg/mL anti-human IgG (Jaxon Immuno., Cat. No. 109–005-003). – Samples were diluted serially 1:3 starting at 1:300 and ending at 1:8100. Standard IgG curves were also included in total antibody ELISAs using IgG isolated from human serum (Sigma Aldrich, Cat. No. 14506–10MG).

### Anti-His tag ELISA

An anti-His tag ELISA was used to determine if there was a difference in binding efficiency of the separate RBDs to the 96 well plates used in all respective ELISAs. One half of a 96 well plate was coated with 1 µg/mL Wuhan-Hu-1 RBD and the other half with 1 µg/mL Omicron BA.4/BA.5 RBD. Initial incubation and blocking steps were identical to SARS-CoV-2 RBD ELISAs. HRP conjugated anti-his tag Antibody (ACROBiosystems, Cat. No. HIS-LY63) was added to both RBDs in triplicates diluted serially 1:4 starting from a 400 µg/mL stock solution. Anti-his tag antibodies were incubated for 2 hours, the plate was washed, and then 50 µL HRP conjugated anti-mouse IgG (Jaxon Immuno., Cat. No. 115–035-166) at a 1:1000 dilution in TBST was added to each well to amplify signal. The plate was incubated for 1 hour, washed, and then substrate and absorbance recording steps were performed as described above.

### SARS-CoV-2 neutralization assays

Wuhan-Hu-1 and Omicron BA.4/BA.5 neutralization assays were carried out in parallel. A series of dilutions of blood/DMEM eluates were tailored to the time interval prior to or post bivalent boost, allowing for an accurate NT50 to be generated. All samples were run in duplicate. For saliva assays, samples were added undiluted followed by a 1:2 serial dilution in DMEM in the following 4 wells for all timepoints.

A positive and negative control was included in every assay. The negative control was created by manually adding Normal Human Donor Serum (Saturn Biomedical, Cat. No. 320927835) collected pre-pandemic to washed whole blood cells, donated from University of Massachusetts Health Services. The mixture was then used to create DBS and blood eluates for future assay use. The positive control was a DBS donation in which a participant gave additional donations at the Post 1 timepoint. True controls for saliva were not included due to the inability to acquire saliva donated before 2019 and the volume of saliva it would take for a positive control to be included in all assays. Instead, wells containing no saliva were used as a negative control and wells that received no pseudo-virus were used as positive controls. All samples and controls consisted of 40 µL volumes. Pseudo-virus aliquots were diluted to deliver 250 infective units per well in 40 µL DMEM. Both Wuhan and Omicron BA.4/BA.5 pseudo-viruses contained the gene encoding firefly luciferase. Plates were incubated at 37°C for 1 hour with plate swirling every 15 minutes. 20,000 HEK293T cells expressing ACE2 were then added to each well in 40 µL DMEM (5x10^5^ cells/ml) with 5 µg/mL polybrene as a transfection agent. Plates were incubated for 72 hours at 37°C. Following transduction, the wells received 120 µL lysis buffer (20 mM Tris-HCl, 100 µM EDTA, 1.07 mM MgCl _2_, 2.67 mM MgSO _4_, 17 mM dithiothreitol (DTT), 250 µM ATP and 250 µM D-luciferin, 1% Triton-X) and were shaken for five minutes and luciferase activity recorded using a Spectramax L luminometer (Molecular Devices).

### Determination of SARS-CoV-2 variants in the test population

Variant percentages present on the UMass campus were evaluated using whole-genome sequencing (WGS). Anterior nasal (AN) swab samples collected from individuals suspected of infection with SARS-CoV-2 were first tested using the ICTC SARS-CoV-2 qPCR clinical assay. Samples returning a result of “positive” and that met QC criteria were prepared and sent for WGS at Ginkgo Bioworks (Boston, MA). Variant percentages were then calculated based on the WGS results. Note that these percentages were derived from results of AN swab samples submitted for voluntary, symptomatic testing, and are not a true representation of all circulating SARS-CoV-2 variants present in the community at each given timepoint.

### Statistical analysis

GraphPad Prism 10 was used for the calculation of antibody concentrations. Standard curves were interpolated as sigmoidal, four parameter, X is concentration. For comparison of titers, data were assumed to have a non-Gaussian distribution, and comparisons made using Wilcoxon matched-pairs signed rank test and the Mann-Whitney test with geometric mean titers (GMT). No adjustments were made for multiple-testing.

Neutralization assay NT50 values were generated by analyzing Percent Neutralization Values above 0 by non-linear regression log(inhibitor) vs. normalized response—Variable Slope Least Squares fit using GraphPad Prism as described in Ferrera and Temperton (15).

All data sets were analyzed in GraphPad Prism 10. For all tests, p < 0.05 was considered statistically significant, and all tests were two-tailed.

Comparison of the titers between the normalized and unnormalized methods was via quartile (median) regression reported with adjusted R^2^ (16, 17).

## Results

### Demographics

**Figure 1:**
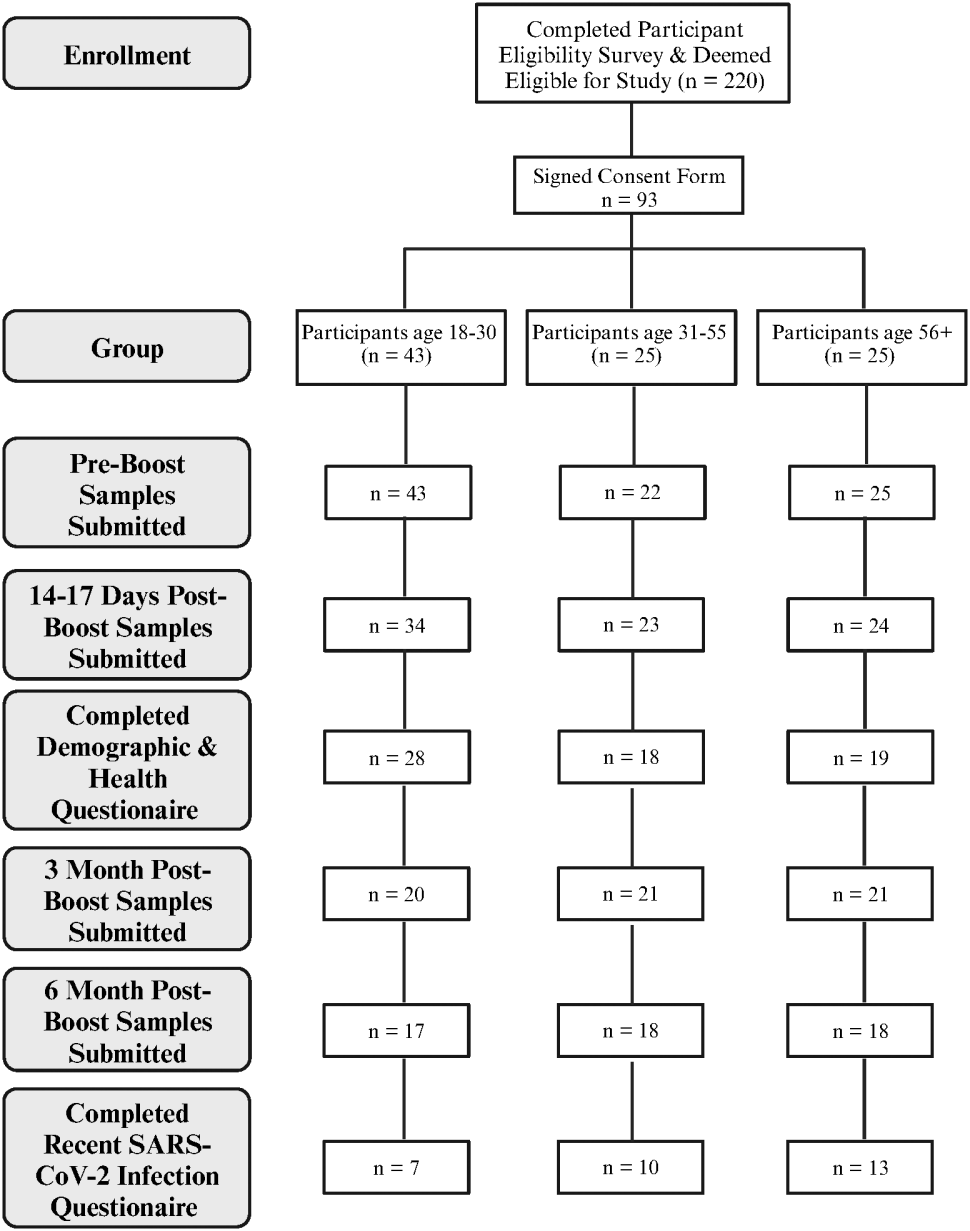
Overview of participant enrollment and sample collection. Created with BioRender.com.

As shown in **Figure 1**, 220 people who completed the initial screening survey were eligible to participate. Of these, 93 completed the informed consent and provided pre-boost samples. Of this total, 80 participants provided both the pre-boost and the 2–3 weeks post-boost samples. One individual did not provide pre-boost donations but provided samples at all future timepoints. Of these 81 participants that provided the Post 1 donation, 62 participants provided samples at 3 months post-boost, and 53 of these also provided samples at 6 months post-boost. A similar number of participants among the 3 age groups provided samples at 3- and 6-months post boost.

Demographic data for the 81 participants who provided post-boost samples are shown in **Table 1**. Participants reported their age by broad category, with the majority (72.8%) being <55 years old. Of the participants, 53.1% identified as female and 27.2% male. Most participants identified as White (64.2%) or Asian (13.6%).

**Table 1:** Demographic information of participants.

N=81	
Characteristic	n (%)
Age	
18–30	35(43.2)
31–55	24(29.6)
56+	22(27.2)
Sex	
Female	43(53.1)
Male	22(27.2)
No response/prefer not to answer	16(19.8)
Self-Identification	
White	52(64.2)
Asian	11(13.6)
Hispanic	1(1.2)
No response/prefer not to answer	17(21)
Number of Previous SARS-CoV-2 Infections	
0	20(24.7)
1	36(44.4)
2	7(8.6)
3	1(1.2)
No response/prefer not to answer	17(21)
Bivalent BA.4/BA.5 Booster Received	
Moderna	43(53.1)
Pfizer/BioNTech	21(25.9)
Unspecified	17(21)
Initial SARS-CoV-2 Vaccination	
Moderna	33(40.8)
Pfizer/BioNTech	25(30.9)
Johnson & Johnson	3(3.7)
Other	3(3.7)
No response/prefer not to answer	17(21)
Previous SARS-CoV-2 Boosters	
1	38(46.9)
2+	26(32.1)
No response/prefer not to answer	17(21)
First SARS-CoV-2 Booster	
Moderna	46(57.5)
Pfizer/BioNTech	18(22.5)
Second SARS-CoV-2 Booster	
Moderna	11(13.75)
Pfizer/BioNTech	13(16.25)
Third SARS-CoV-2 Booster	
Moderna	2(2.5)
Pfizer/BioNTech	0(0)

Previous reported infections with SARS-CoV-2 ranged from no known prior infection (24.7% of participants) to three previous infections (1 participant), with a single previous infection being the most frequent exposure (44.4%). Twenty-one percent (n=17) participants provided no information regarding previous infections. Similarly, 21% of participants provided no information on previous SARS-CoV-2 initial vaccination or previous monovalent boost, while the majority of participants (79%), were vaccinated and had 1–3 previous monovalent boosts. About half of the participants (53.1%) reported receiving the bivalent boost manufactured by Moderna, while 25.9% reported receiving the bivalent boost manufactured by Pfizer/BioNTech, and the remaining 21% did not answer this question. With respect to infection after receiving the boost, 6 participants reported testing positive for COVID-19 between the 3-month and 6-month post-boost timepoints and 1 participant tested positive between the 2–3 week –day and 3-month timepoints. Additionally, with the use of our questionnaire, 8 participants were identified to have previously tested positive for COVID-19 between April 2022 and June 2022 when Omicron BA.4/BA.5 was the dominant circulating variant.

### RBD binding and virus neutralization increases after boost

IgG antibody levels with the ability to bind the Wuhan-Hu-1 RBD and/or Omicron BA.4/BA.5 RBDs, measured via ELISA, as well as neutralizing ability against Wuhan-Hu-1 and Omicron BA.4/BA.5 pseudo-viruses, measured via cell-based neutralization assay, were assessed in the blood and saliva longitudinally over the 6-month period. Absorbance values within the linear region of the standard curves (**Supplementary Figure S1**) were interpolated and multiplied by the dilution factor to determine the concentration of antibodies in samples. Concentrations are indicative of antibodies in the resuspended blood eluates, not circulating concentrations in human sera. Because a standard curve for Omicron BA.4/BA.5 RBD was not available, the standard curve for Wuhan-Hu-1 RBD binding antibodies was used to calculate concentrations of Omicron BA.4/BA.5 RBD binding antibodies. We validated this approach by measuring the RBD binding efficiencies of both Wuhan-Hu-1 and Omicron BA.4/BA.5 RBDs to the 96-well plates via a HIS-tag ELISA (**Supplementary Figure S2**). The levels of 50% neutralizing titer (NT50) were calculated from results of the neutralization assays.

**Figure 2:**
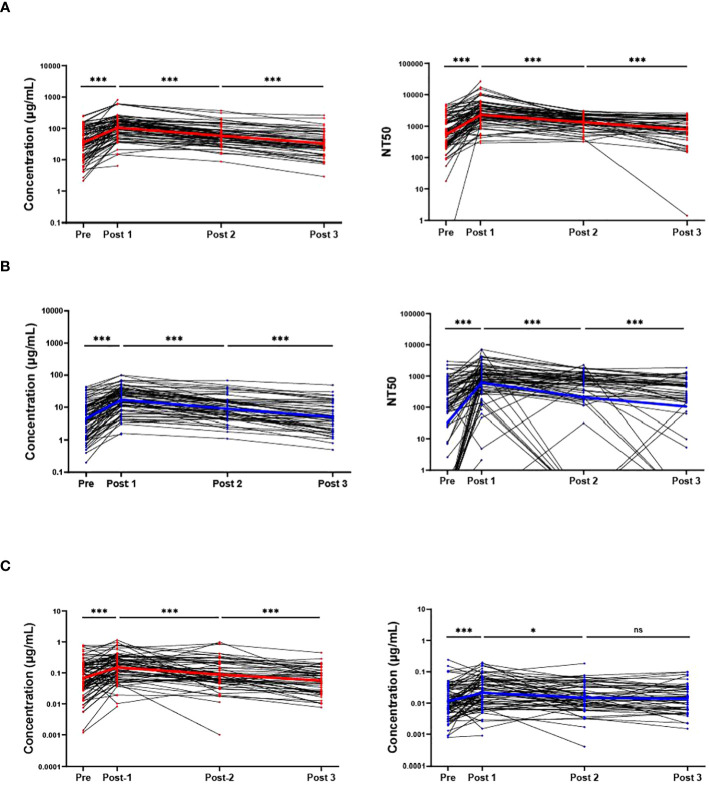
RBD binding and virus neutralization increases after boost. Antibody responses against ancestral Wuhan-Hu-1 strain (red) and the Omicron BA.4/BA.5 variant (blue) are shown. Individual IgG concentrations and 50% neutralizing titer (NT50) detected in blood eluates against Wuhan-Hu-1 pseudo-virus **(A)**, and against the Omicron BA.4/BA.5 variant **(B)**. The IgG levels from saliva also are shown against both variants **(C)**. Solid colored lines represent geometric means in the respective sample set of each timepoint. Sample populations are as follows:to Pre N=80, Post 1 N=81, Post 2 n=61, Post 3 n=46. ns, not significant; * <0.05; ***p <0.001; Wilcoxon matched-pairs signed rank test assuming non-Gaussian distribution.

In blood eluates of the sample population the anti-Wuhan IgG concentration showed a statistically significant increase 2–3 weeks after boost from a mean of 35 µg/mL to a mean of 104 µg/mL (p<0.001). This concentration also statistically significantly decreased at later timepoints to 58 µg/mL (Post 1 to Post 2, p<0.001), and 33 µg/mL (Post 2 to Post 3, p<0.001) respectively. The NT50s of the Wuhan-Hu-1 pseudo-virus also showed the same trends, with a statistically significant difference in the means of 546, 2277, 1340, and 774 for the respective timepoints (**Figure 2A**). Interestingly, but not surprisingly, the participant with the lowest anti-Wuhan IgG concentration at Post 3 also had the lowest NT50 at Post 3, and another participant who showed no neutralizing ability at Pre and Post 1 had the lowest anti-Wuhan IgG concentration at Post 1 (**Figure 2A**). Similar to the anti-Wuhan IgG, the anti-Omicron IgG from blood eluates showed a statistically significant increase 2–3 weeks after boost, from a mean of 4.5 µg/mL to a mean of 17.1 µg/mL (p<0.001) with a decrease at later timepoints with means of 9.0 µg/mL (Post 1 to Post 2, p<0.001), and 5.0 µg/mL (Post 2 to Post 3, p<0.001), respectively. The NT50s of the Omicron BA.4/BA.5 pseudo-virus also followed the same trend, increasing from Pre to Post 1 with means of 32 and 617 (p<0.001) and decreasing to 207 (Post 1 to Post 2, p<0.001) and 105 (Post 2 to Post 3, p<0.001) for the respective timepoints. Several samples were identified as non-neutralizing and were assigned an NT50 value of 0.1 for analysis (**Figure 2B**).

Similar to IgG levels obtained from blood eluates, the overall participant IgG levels (both anti-Wuhan and anti-Omicron) in saliva also increased 2–3-weeks post-boost and decreased at later timepoints with a slight difference that did not reach statistical significance between anti-Omicron IgG obtained from saliva between the 3-month (Post 2) and 6-month (Post 3) timepoints (p = 0.38). The means of the anti-Wuhan IgG obtained from saliva are 0.065 µg/mL, 0.150 µg/mL, 0.088 µg/mL, and 0.57 µg/mL, while the means for anti-Omicron IgG from saliva are 0.011 µg/mL, 0.021 µg/mL, 0.015 µg/mL, and 0.014 µg/mL (**Figure 2C**). The neutralizing ability of saliva against both Wuhan-Hu-1 pseudo-virus and Omicron BA.4/BA.5 pseudo-virus also increased significantly 2–3-weeks post-boost however, a significant decrease was only detected between Pre and Post 2 for saliva against Wuhan-Hu-1 pseudo-virus with no statistically significant differences detected at the later timepoints against the Omicron BA.4/BA.5 pseudo-virus (**Supplementary Figure S3**). SIgA specific to the two variants was also measured in the saliva via ELISA over the timepoints but no significant changes detected with our methods and no conclusions were made from these data (**Supplementary Figure S4**). It is important to note that **Figure 2** does not include sample results from individuals who tested positive for COVID-19 post-bivalent boost within the 6-month timeframe. Wuhan-Hu-1 IgG levels in the saliva were correlated with IgG levels obtained from blood eluates via quartile (median) regression. The correlation was significant but weak with an r^2^ value of 0.101 (**Supplementary Figure S5**). A portion of saliva samples that had sufficient aliquots remaining after completing all SARS-CoV-2 specific assays were used in total antibody ELISAs to normalize for immunoglobulins in saliva samples (Wuhan-Hu-1 Ig/total-Ig) and again correlated with the antibody levels received from DBS. A stronger correlation is suggested by the new r^2^ value of 0.323 (**Supplementary Figure S6**).

There were 8 individuals in our study who previously tested positive for COVID-19 between April and July of 2022, prior to the bivalent boost. These individuals were expected to have been infected with either the Omicron BA.4 or BA.5 variant due to these variants being the dominant variants found on our campus at this time. These individuals, on average, had higher anti-Omicron antibody concentrations and NT50s than the rest of the sample population, with geometric means 12.0 µg/mL and 408 for Pre, 33.2 µg/mL and 1573 for Post 1, 11.4 µg/mL and 893 for Post 2, and 8.3 µg/mL and 646 for Post 3. However, several individuals had higher Omicron BA.4/BA.5 specific response who did not test positive for COVID-19 around this time frame. Additionally, these 8 individuals who likely had an Omicron BA.4/BA.5 infection, also had higher geometric means for Wuhan specific responses than the average population. With concentration and NT50 geometric means of 115.3 µg/mL and 1545 for Pre, 246.1 µg/mL and 5472 for Post 1, 94.7 µg/mL and 2126 for Post 2, and 57.7 µg/mL and 1163 for Post 3.

### Wuhan-Hu-1 antibody response remains higher than Omicron BA.4/BA.5

**Figure 3:**
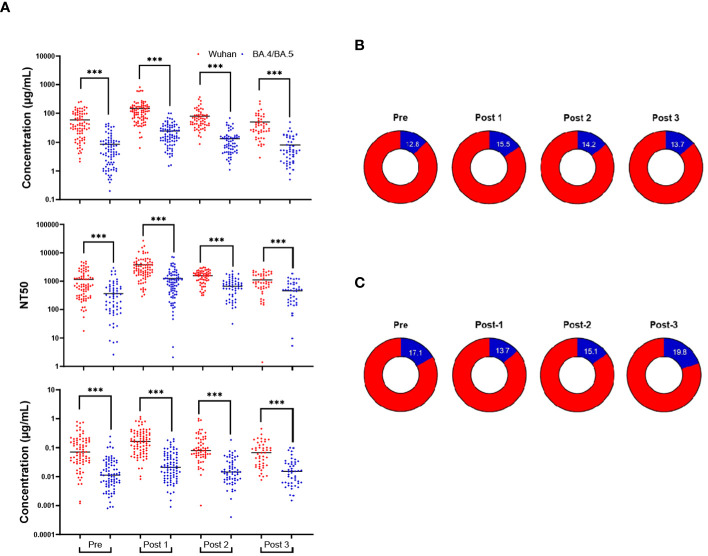
Wuhan-Hu-1 antibody response is greater than that of Omicron BA.4/BA.5. Comparison of the antibody response to the Wuhan-Hu-1 strain with the Omicron variant **(A)**: IgG concentrations in DBS eluates (upper), NT50s of DBS eluates (middle), and IgG concentrations in saliva (lower). The percentage of the anti-Omicron BA.4/BA.5 IgG (blue) from the total-RBD-IgG in blood eluates **(B)** and saliva **(C)**. ***p<0.001; Mann-Whitney test.

Across all immunoassays, the IgG response against Wuhan-Hu-1 was much higher than the response against Omicron BA.4/BA.5. For IgG obtained from blood eluates, NT50 from blood eluates and IgG obtained from saliva, the Wuhan-Hu-1 response was consistently greater than the response against Omicron BA.4/BA.5 (**Figure 3A**). Antibody concentrations of a given timepoint (both Wuhan-Hu-1 IgG and Omicron BA.4/BA.5) in blood eluates were aggregated to create a total-RBD-IgG at each timepoint for each participant. The Omicron BA.4/BA.5 specific IgG of that respective timepoint was then normalized by the total-RBD-IgG to give an arbitrary percentage of total-RBD-IgG that has the ability to bind Omicron BA.4/BA.5 RBD. This percentage of Omicron BA.4/BA.5 specific IgG of the total measured RBD-IgG was much more stable and did not display large fluctuations in the population between the timepoints (**Figure 3B**). Parallel analyses with antibodies obtained in the saliva show broadly similar trends (**Figure 3C**). It is important to note that **Figure 3** does not include results from individuals who tested positive for COVID-19 (SARS-CoV-2 infection) post-bivalent boost within the 6-month time frame.

### Antibody responses in individuals testing positive for COVID-19 post-bivalent boost

**Figure 4:**
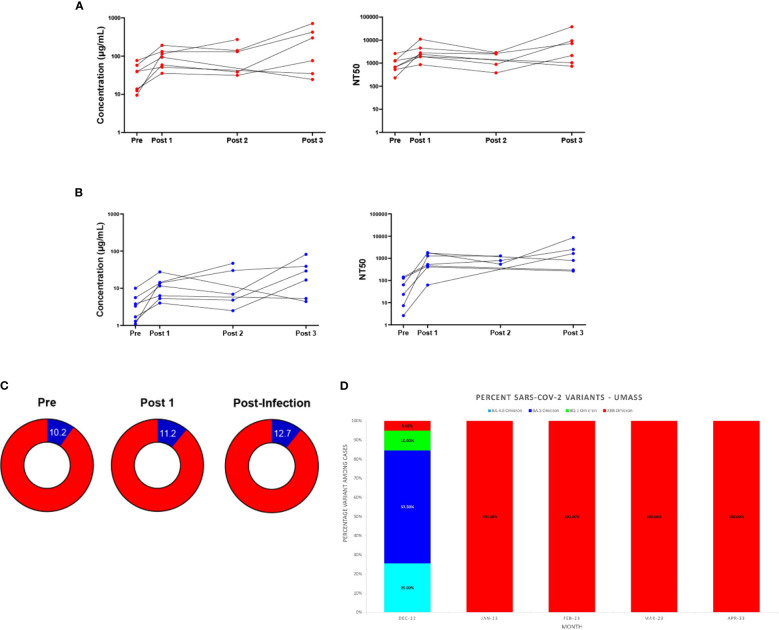
Antibody responses in individuals testing positive for COVID-19 after the bivalent boost provide support for immune imprinting. Antibody (IgG) responses measured in DBS eluates against ancestral Wuhan-Hu-1 strain **(A)** and Omicron BA.4/BA.5 strain **(B)** for the seven individuals who tested positive for COVID-19 after receiving the bivalent boost. Individual IgG changes over time (left) and levels of 50% neutralizing titer (NT50) against respective pseudo-virus (right) are shown. Sample populations are as follows: Pre & Post 1 n=7, Post 2 n=5, Post 3 n=6. Part of whole graphs **(C)** show percentage of anti-Omicron BA.4/BA.5 IgG (blue) in blood eluate out of total-RBD-IgG in individuals testing positive for COVID-19 post-bivalent boost. **(D)** The XBB Omicron variant replaced BA.5 Omicron as the most prevalent SARS-CoV-2 variant on the UMass, Amherst, Campus beginning in January 2023. SARS-CoV-2 variant percentages calculated from whole-genome sequencing (WGS) results are shown.

Individuals who tested positive for COVID-19 post-bivalent boost were analyzed separately from those remaining infection-free after the bivalent boost. Seven individuals fit these criteria, with 6 individuals testing positive between the 3-month (Post 2) and 6-month (Post 3) timepoints and 1 individual testing positive between the 2–3-week (Post 1) and 3-month (Post 2) timepoints. We note that the individual who tested positive between the 2–3-week and 3-month timepoints did not donate a 6-month sample. Additionally, two individuals who tested positive between the 3-month and 6-month timepoints did not donate 3-month (Post 2) samples. For 5 of the 7 individuals, both Wuhan-hu-1 IgG and Omicron BA.4/BA.5 levels in blood eluates increased at the timepoint after infection and these levels exceeded their IgG levels 2–3-weeks post-boost (**Figures 4A, B**). Similar trends are also seen with the analyses of IgG in saliva, with 4 of the 7 individuals increasing in both IgG at the timepoint after infection which exceeded IgG values 2–3-week post-boost (**Supplementary Figure S5**). The seven individuals who experienced an infection after receiving the bivalent boost were also analyzed separately from the total study population to assess if the additional exposure to a current strain of SARS-CoV-2 stimulated an increase in the percentage of Omicron BA.4/BA.5 specific antibodies. Timepoints Pre, Post 1 and the timepoint after immediately following individual infections (Post Infection) were analyzed. These individuals did not have a statistically significant change in the percentage of Omicron BA.4/BA.5 IgG after receiving the boost, nor did the percentage change after the most recent infection (**Figure 4C**). Interestingly, all of these participants tested positive for COVID-19 in the first quarter of 2023 (January 1^st^ through March 31^st^). With the use of data donated from the IALS Clinical Testing Center (ICTC) at the University of Massachusetts Amherst, which tested the local population for COVID-19 on a regular basis, these participants were likely to all have been infected with the SARS-CoV-2 variant XBB.1.5 Omicron (**Figure 4D**).

## Discussion

Across a diverse set of participants, we found evidence for a statistically significant increase in antibodies specific for both SARS-CoV-2 variants after a single dose of the bivalent boost, although the response to the Wuhan-Hu-1 variant was much stronger than response to the Omicron BA.4/BA.5 variant at all timepoints. This supports the findings of previous studies that a single dose of this bivalent booster does not produce a strong antibody response specific to the Omicron BA.4/BA.5 spike protein (5–9). Our data from the DBS ELISA, DBS neutralization assays, as well as saliva ELISAs were consistent and revealed that the Wuhan-Hu-1 antibody response dwarfed the Omicron BA.4/BA.5-specific response in this population before the boost, at all timepoints after the boost, and in individuals who became infected after receiving the bivalent boost. Based on our methods, it is possible to estimate that the amount of IgG with the ability to bind these newer variants remains proportional to the Wuhan-Hu-1 wildtype throughout all timepoints, and that the BA.4/BA.5 component of the vaccine did not elicit a specific immune response. These data could possibly be explained by the occurrence of immune imprinting, also known as original antigenic sin. Original antigenic sin was first proposed in 1960 studying individuals immunized against influenza, and has been well-documented in dengue pathogenesis (18). This process, now referred to as immune imprinting, can best be described as an immune response to an initial exposure that imprints itself on the immune system, altering the responses to subsequent infections and vaccinations (19–22). It is now apparent from recent publications that immune imprinting also occurs with SARS-CoV-2: serum responses derived primarily from B cells that developed after an initial exposure overwhelmed new antibody responses from naïve B cells (21–27). A recent publication also indicated that the incorporation of the mRNA encoding the wildtype spike protein in this bivalent boost results in a deep immunological imprinting and the authors recommend its removal from future vaccinations (26). Our study cannot prove this theory, but it does support other studies that describe imprinting in the SARS-CoV-2 response (20–26). Additionally, a definitive analysis is difficult given that individuals greatly vary in infection and/or vaccination history, which can affect their immune responses to different SARS-CoV-2 variants in the future (28).

When considering our saliva data, it was both noteworthy and reassuring to see the trend of the Wuhan-Hu-1 antibody response dominating the Omicron BA.4/BA.5 response in these samples, similar to that observed in our blood eluate samples. This finding alone supports the use of saliva in studies to analyze population trends. However, even though the IgG in the blood was significantly correlated with the IgG obtained from the saliva, the correlation became stronger when saliva samples were normalized to total antibodies, in agreement with a previous publication (29). If the goal of examining antibody concentrations in the saliva is to predict circulation levels, then proper normalization is necessary. As the primary antibody response is specific to the Wuhan-Hu-1 wildtype, this is likely the reason our saliva assays were not sensitive enough to detect as many significant changes in Omicron BA.4/BA.5-specific antibodies as compared to the Wuhan-Hu-1-specific antibodies. It also was interesting to find no secretory IgA trends over time in response to this bivalent boost, given that previous studies have found mucosal responses in individuals fitting certain criteria such as hybrid immunity (29, 30). Normalization of the entire saliva data set may have provided a more accurate analysis of the mucosal antibody response, unfortunately we were limited by the volume of the saliva samples. Since the amount of saliva produced by a participant in a short amount of time is often limited, future studies should encourage participants to collect saliva over a longer time period, e.g., 30 minutes and set a minimum volume of 10 mL.

It is important to note that this study only examined the antibody response after the 2022 bivalent boost. Thus, our study only considered a small portion of the adaptive immune response and did not consider the cell-mediated immune response.

Overall, we present a comprehensive data set for the antibody response of eighty-one individuals who received the SARS-CoV-2 bivalent boost. The majority of these individuals gave samples over a six-month time period following the administration of the boost, and a sub-population of participants tested positive for COVID-19 after receiving the bivalent boost, while still giving samples after the positive test. In both blood and saliva, the antibody response against the original wildtype (Wuhan-Hu-1) variant remains much stronger than the response against the Omicron BA.4/BA.5 variant.

This study included a diverse cohort with excellent follow up, however, several limitations were identified. This study was not powered to make comparisons with a small number of participants in sub-studies. The majority of the participants in this study were affiliated with the university and may not be a great representation of the general population. There is also the potential for unreported subclinical/asymptomatic infections.

## Data availability statement

The original contributions presented in the study are included in the article/**Supplementary Material**. Further inquiries can be directed to the corresponding authors.

## Ethics statement

The studies involving humans were approved by UMass-Amherst IRB # 3852, October 11, 2022. The studies were conducted in accordance with the local legislation and institutional requirements. The participants provided their written informed consent to participate in this study.

## Author contributions

RB: Data curation, Formal analysis, Investigation, Methodology, Writing – original draft, Writing – review & editing. RL: Data curation, Investigation, Methodology, Writing – original draft. MS: Data curation, Methodology, Software, Writing – original draft. JP: Data curation, Software, Writing – original draft. AE: Data curation, Methodology, Writing – original draft. AL: Conceptualization, Data curation, Formal analysis, Methodology, Supervision, Validation, Writing – original draft, Writing – review & editing. DA: Conceptualization, Formal analysis, Methodology, Writing – original draft, Writing – review & editing. PR: Conceptualization, Data curation, Formal analysis, Funding acquisition, Supervision, Writing – original draft, Writing – review & editing. KA: Conceptualization, Data curation, Methodology, Supervision, Writing – original draft, Writing – review & editing. BO: Conceptualization, Funding acquisition, Supervision, Writing – original draft, Writing – review & editing.

## Footnotes

1 Pfizer-BioNTech COVID-19 Vaccine, Bivalent (Original and Omicron BA.4/BA.5) | Official HCP Site | Risk Info (2023). https://www.cvdvaccine-us.com/ [Accessed August 15, 2023]

2 COVID-19 Vaccine, Bivalent | Moderna | For Vaccine Recipients (2023). https://eua.modernatx.com/recipients?tc=ps_qevnlq9&cc=4003&utm_source=google&utm_medium=search&utm_campaign=bnd+booster&gclsrc=aw.ds&&gclid=CjwKCAjwxOymBhAFEiwAnodBLJJfl_hD8YVcf9oIouBgtafHsJzC0edQiDQh754dHjLvtgQeRGlE8RoC9_sQAvD_BwE [Accessed August 15, 2023]
