# Supplementary material for: Antibody responses in blood and saliva post COVID-19 bivalent booster do not reveal an Omicron BA.4/BA.5- specific response
Source: Front Immunol. 2024 May 15;15:1401209. doi: 10.3389/fimmu.2024.1401209 (PMC11133519; doi:10.3389/fimmu.2024.1401209)
Supplement: Supplementary file 1 [file DataSheet_1.pdf]

Supplementary Material

Figure S1

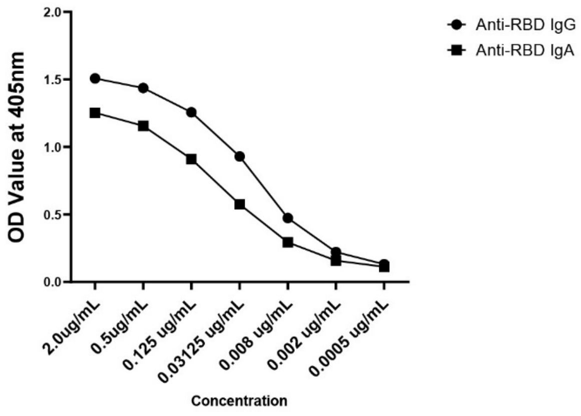

Figure S1) Average of standard curves generated from all ELISAs ran for IgG binding against Wuhan-Hu-1 RBD. Highest concentrations of standard antibodies began and 2.0ug/mL and were diluted 1:4 serially in the following 6 wells. All results recorded in OD at 405nm.

Figure S2

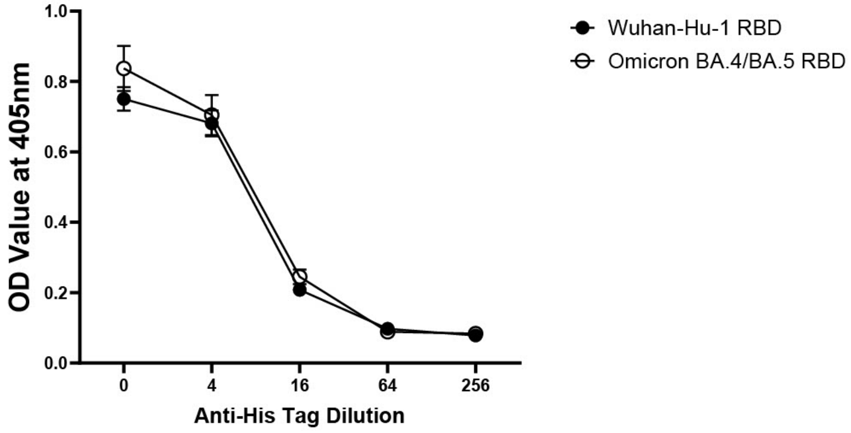

Figure S2) RBD binding efficiencies of the two RBDs to the 96-well plates via a HIS-tag ELISA. Curves were generated diluting anti-his tag IgG 1:4 serially and measuring OD value at 405nm.

Figure S3

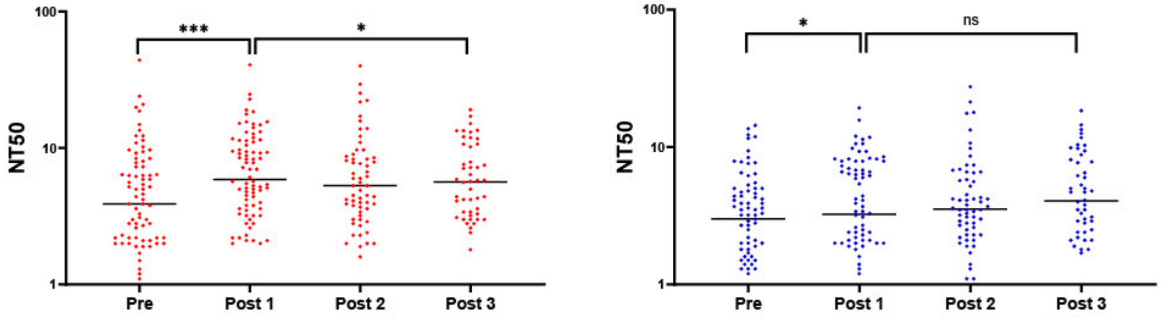

Figure S3) NT50s measured from saliva against Wuhan-Hu-1 pseudoviurs (left) and Omicron BA.4/BA.5 pseudovirus (right) across all timepoints. *** represents p<0.001 and * represents p<0.05 between respective timepoints after non-parametric Mann-Whitney test (unpaired).

Figure S4

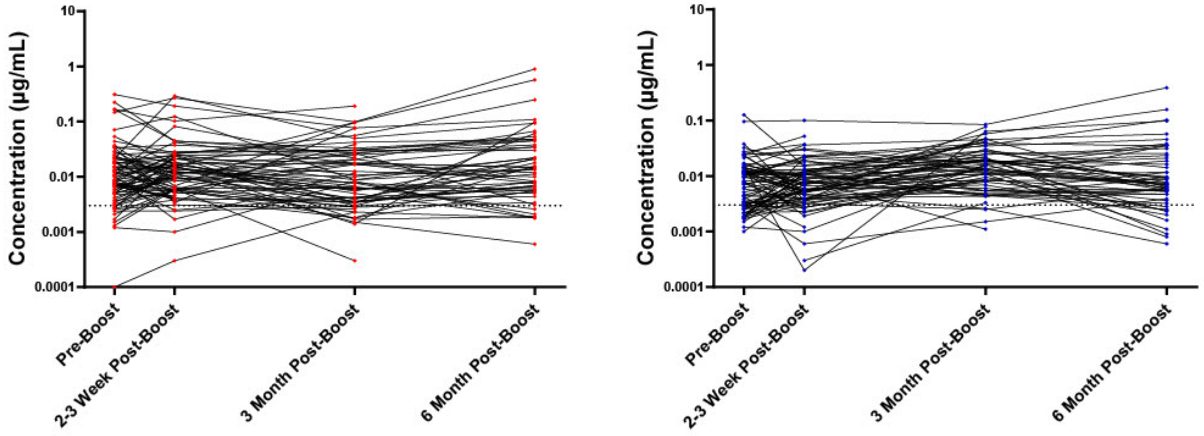

Figure S4) IgA measured from saliva against Wuhan-Hu-1 RBD (Left) and Omicron BA.4/BA.5 RBD (right). Graphs depict individual IgA changes over time. The horizontal dotted line indicates the positive cutoff of 3x ELISA background interpolated on standard curve. Sample populations are as follows; Pre & Post 1 n=80, Post 2 n=61, Post 3 n=46. No significant changes were detected between respective timepoints after non-parametric Wilcoxon matched-pairs signed rank test assumed to have non-Gaussian distribution.

Figure S5

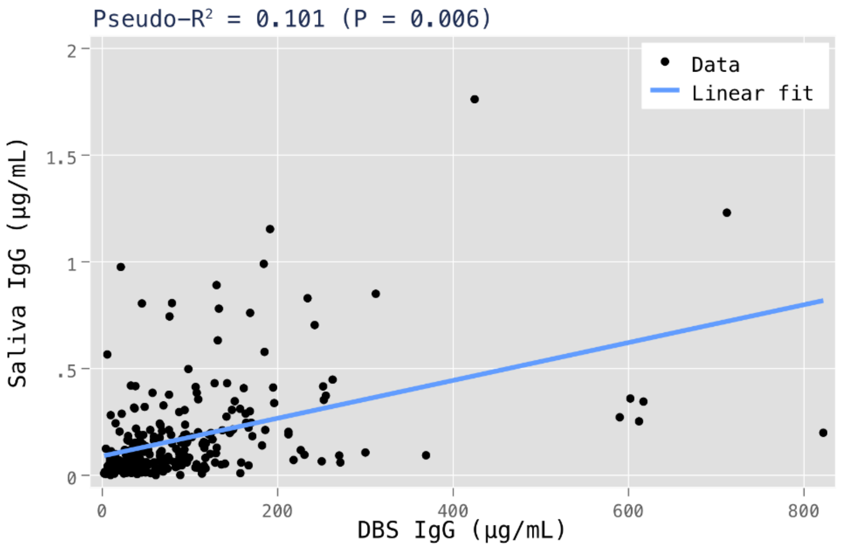

Figure S5) Median regression of anti-Wuhan-IgG levels obtained from saliva and DBS.

Figure S6

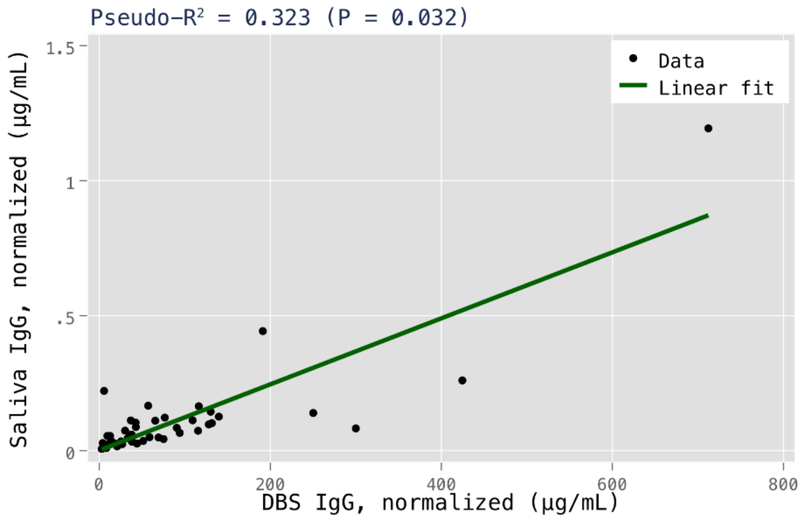

Figure S6) Median regression of anti-Wuhan-IgG levels obtained from saliva normalized by dividing by total IgG obtained from respective saliva samples and anti-Wuhan-IgG from DBS.

Figure S7

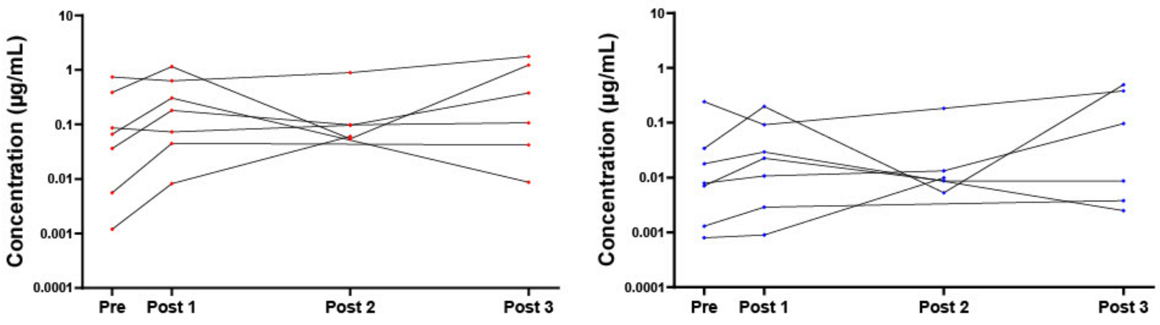

Figure S7) IgG measured from saliva against Wuhan-Hu-1 RBD (Left) and Omicron BA.4/BA.5 RBD (right) from individuals testing positive post-bivalent boost. Graphs depict individual IgG changes over time.
